# A survey of community pharmacists’ perceptions, attitudes, and practices regarding support for physical activity, exercise, and frailty

**DOI:** 10.1186/s40780-026-00588-w

**Published:** 2026-05-28

**Authors:** Takuya Azechi, Miho Yamamura, Eiji Kose, Toshimi Kimura

**Affiliations:** 1 Faculty of Pharmacy, Juntendo University, 6-8-1 Hinode, Urayasu, Chiba 279-0013 Japan; 2 Department of Pharmacy, Juntendo University Hospital, 3-1-3 Hongo, Bunkyo-ku, Tokyo, Japan

**Keywords:** Community pharmacist, Frailty, Physical activity, Exercise, Older adults

## Abstract

**Background:**

Community pharmacists are expected to contribute to frailty prevention through physical activity and exercise support. However, their definitive involvement and the factors influencing their engagement remain unclear.

**Methods:**

An anonymous online questionnaire-based survey targeting pharmacists working in community pharmacies or the pharmacy sections of drugstores was conducted from November 2024 to March 2025. The survey assessed their recognition, attitudes, and practices regarding frailty and support for physical activity and exercise. Frailty assessment was defined as responses of “occasionally” or more. Descriptive statistics and multivariate logistic regression were used for the analysis.

**Results:**

Among the 1,054 responses, 89.8% expressed positive attitudes toward supporting physical activity and exercise, but insufficient knowledge and lack of confidence were major barriers. Furthermore, although 88.3% of respondents considered awareness of the patient frailty status to be important and 60.1% recognized the importance of pharmacist-led frailty assessments, only 29.6% performed such assessments. The independent factors associated with frailty assessment were implementation of pharmacist-led support for physical activity and exercise (adjusted odds ratio [aOR]: 2.46; 95% confidence interval [CI]: 1.73–3.50), recognition of the importance of frailty assessment (aOR: 2.83; 95% CI: 1.97–4.07), and availability of educational opportunities related to physical activity and exercise support (aOR: 1.62; 95% CI: 1.12–2.34).

**Conclusion:**

While many pharmacists recognize the importance of frailty care, their involvement remains limited due to insufficient knowledge. Practical and educational experiences regarding physical activity and exercise support were significantly associated with frailty assessment practices, suggesting their importance in promoting future pharmacists’ engagement in frailty prevention.

**Supplementary Information:**

The online version contains supplementary material available at 10.1186/s40780-026-00588-w.

## Background

Frailty and sarcopenia are key barriers to a healthy life expectancy among older adults. Frailty refers to a state of increased vulnerability to stressors due to an age-related decline in physiological functions, leading to a higher risk of adverse health outcomes [1, 2]. In Japan, frailty is recognized as a pre-disability state between robust health and the need for long-term care; thus, early intervention for its prevention is strongly emphasized [3]. Sarcopenia, an age-associated decline in muscle mass, strength, and physical performance that compromises gait and balance, is a core component of frailty [4–6]. Both frailty and sarcopenia increase the likelihood of falls, dependency, and mortality [6, 7]. However, these conditions are preventable and reversible through appropriate interventions. In particular, physical activity and exercise can increase muscle mass and strength, thereby improving mobility and activities of daily living and contributing to the prevention and mitigation of frailty and sarcopenia. These efforts are expected to help maintain and improve the quality of life and longevity of older adults.

In Japan, community pharmacies and drugstores with a pharmacy section are essential components of the community-based integrated-care system and are expected to play a role in disease prevention and health promotion in the general population [8]. Pharmacists in these settings are in a unique position to support the health behaviors of community residents through health consultations and recommendations to seek medical care when necessary. In recent years, healthcare providers, including pharmacists, have been called upon to deliver personalized messages that encourage physical activity based on individual readiness [9]. Guidelines also recommend appropriate types and amounts of exercise, particularly for older adults and individuals with chronic conditions [9, 10]. Moreover, given that polypharmacy and the number or type of medications taken are associated with an increased risk of falls, pharmacists should support physical activity with consideration of medication history, thus contributing to frailty and sarcopenia prevention [11].

Through their provision of physical activity support and frailty assessment, community pharmacists can help maintain the health of older adults and ensure safe pharmacotherapy. Evidence suggests that pharmacist-provided information on exercise may be useful in delaying the progression of frailty in patients [12]. In some cases, pharmacists conduct simplified frailty assessments at the pharmacy, offer guidance on frailty prevention, and communicate their findings to physicians [13]. Furthermore, frailty has been linked to polypharmacy and adverse drug events, suggesting that pharmacist-led frailty assessment may also support safer medication management [14–17]. The Asia-Pacific Clinical Practice Guidelines for the Management of Frailty specifically recommend de-prescribing potentially inappropriate medications to address polypharmacy, indicating the key role that pharmacists play in this domain [18]. Moreover, the concept of rehabilitation pharmacotherapy, which integrates medication management with functional recovery and rehabilitation perspectives, has gained attention in geriatric care [19]. Rehabilitation pharmacotherapy is an approach to medication management that aims to help patients achieve the highest possible levels of physical function, activity, participation, and quality of life. From this perspective, it is important not only to reduce inappropriate medications but also to appropriately assess physical function and adjust pharmacotherapy to maintain or improve functional status. Therefore, from the perspective of rehabilitation pharmacotherapy, pharmacists should assess frailty status, including physical function, and incorporate these assessments into pharmacotherapy.

However, the nationwide status of physical activity support and frailty assessment provided by community pharmacists and the factors that facilitate the implementation of such services have not been sufficiently explored. According to a public opinion survey conducted by the Cabinet Office of Japan, only a small proportion of people consulted pharmacists or pharmacies about exercise habits [20]. Against this background, there is a need to understand the current situation and explore ways to advance pharmacist-led frailty prevention strategies.

Thus, this study aimed to clarify the perceptions, attitudes, and practices of community pharmacists regarding physical activity support and frailty prevention. In particular, we focused on community pharmacists’ experience with conducting frailty assessments and examined the characteristics and associated factors of those who had attempted frailty assessment in practice. To this end, a nationwide survey was conducted to identify the key issues related to the promotion of pharmacist-led frailty interventions.

## Materials and methods

### Study design and participants

In this study, an online cross-sectional questionnaire-based survey was used to investigate community pharmacists’ practices in supporting physical activity and exercise; their perceptions, attitudes, and practices regarding frailty; and their educational experiences and motivation related to physical activity and exercise support. The survey participants were community pharmacists employed in pharmacy chains (dispensing pharmacies and drugstores with a pharmacy section) that operate nationwide in Japan and are committed to community-based healthcare practices, as inferred from their corporate philosophies and management policies. The participating companies agreed to cooperate with the study and distributed the survey invitation to all pharmacists affiliated with their organizations through internal communication channels (e.g., company e-mail or intranet). The total number of pharmacists invited (*n* = 4,997) represents the total number of pharmacists employed by the participating companies at the time of the survey. We did not directly identify or contact individual pharmacists and did not obtain any personally identifiable information. The web link to access the questionnaire was shared through internal communication channels, such as email. The purpose and overview of the study were presented on the questionnaire platform, and informed consent was obtained online. The survey period was from November 1, 2024, to March 31, 2025.

### Questionnaire and measurement scales

An anonymous self-administered questionnaire was used, with survey items carefully designed to ensure that no personally identifiable information was collected. The questionnaire was developed based on a previous study by Malik et al. [21]. The content validity of the questionnaire was reviewed and confirmed by four experts in geriatrics and community pharmacy practice. The details of the questionnaire are provided in Supplemental Table S1. Participants were asked whether they assess frailty in their pharmacy practice. Their responses were categorized into two levels (“Yes: always, sometimes, occasionally” and “No: rarely, never”), which were used as the dependent variable for analysis.

### Data analysis

Data were collected using Questant, an online questionnaire software program (Macromill Inc., Tokyo, Japan). Statistical analysis was performed using JMP Pro 18 (JMP Statistical Discovery LLC, Cary, NC, USA). Descriptive statistics were used to summarize the demographic characteristics of the participants and their responses to each survey item. Cross-tabulation was conducted to compare the pharmacists’ attributes, practices regarding physical activity and exercise support, perceptions and attitudes toward frailty, and experiences and motivation related to education on physical activity, according to whether they reported assessing frailty (“Yes” vs. “No”). The frequency of frailty assessment was measured using a five-point scale (“always,” “sometimes,” “occasionally,” “rarely,” and “never”). For the primary analysis, this variable was dichotomized to identify factors associated with pharmacists who had experience conducting frailty assessments. Pharmacists who responded “always,” “sometimes,” or “occasionally” were classified as having experience with frailty assessment (Yes), whereas those who responded “rarely” or “never” were classified as having no such experience (No). The response option “rarely” was considered to reflect limited or sporadic practice; therefore, it was distinguished from categories indicating at least some degree of practical experience. This classification assumed that there is a significant practical and psychological difference between pharmacists who have never conducted frailty assessments and those who have, even infrequently. Based on this definition, comparisons between two groups were performed using the chi-square test or Fisher’s exact test, as appropriate. In the multivariate logistic regression analysis, frailty assessment in pharmacy practice (Yes vs. No) was used as the dependent variable. Independent variables that showed significant associations with frailty assessment in the univariate analyses were considered for inclusion in the multivariate model. Statistical significance was set at a p-value of less than 0.05.

The free text responses ranged from brief one- or two-word bullet points to more detailed answers containing specific content. Key descriptions that supplemented the quantitative data were quoted directly in the text and presented in italic. In addition, to visualize the response content, a co-occurrence analysis was performed using Text Mining Studio 5.0.2 (NTT DATA Mathematical Systems Inc., Tokyo, Japan). For text preprocessing, synonymous expressions were standardized into a single term; for example, “Kanja,” “Kanja-San,” and “Kanja-Sama” were unified as “Kanja (patient).”

## Results

A total of 1,054 pharmacists responded to the survey, corresponding to a response rate of 21.1%. The demographic characteristics of the respondents are summarized in Table 1, with detailed distributions provided in Supplemental Table S2. Respondents were distributed across all major regional divisions of Japan. Compared with Japanese national statistics [22], the present sample included a relatively higher proportion of pharmacists aged under 40 (Supplemental Table S3). Overall, many respondents reported providing medication counseling to multiple patients daily, and a substantial proportion indicated that a large share of their patients were older adults (Table 1). However, some respondents reported being unaware of the proportion of frail patients within their own patient population (Table 1).

**Table 1:** Characteristics of survey respondents

Characteristics			Frailty assessment in pharmacy practice			
	Overall		No		Yes	
	*N* = 1,054	100.0%	*N* = 742	70.4%	*N* = 312	29.6%
The seven regional divisions						
Hokkaido	156	14.8%	113	15.2%	43	13.8%
Tohoku	204	19.4%	147	19.8%	57	18.3%
Kanto	378	35.9%	260	35.0%	118	37.8%
Chubu	105	10.0%	77	10.4%	28	9.0%
Kinki	169	16.0%	122	16.4%	47	15.1%
Chugoku-Shikoku	21	2.0%	11	1.5%	10	3.2%
Kyusyu	11	1.0%	5	0.7%	6	1.9%
No answer	10	0.9%	7	0.9%	3	1.0%
Pharmacy practice models						
Point-based dispensing pharmacies	531	50.4%	375	50.5%	156	50.0%
Area-based dispensing pharmacies	167	15.8%	110	14.8%	57	18.3%
Drugstore with a pharmacy section	355	33.7%	257	34.6%	98	31.4%
No answer	1	0.1%	0	0.0%	1	0.3%
Number of pharmacists working per day (full-time equivalent)						
<2	391	37.1%	277	37.3%	114	36.5%
2–4	440	41.7%	316	42.6%	124	39.7%
>4	219	20.8%	145	19.5%	74	23.7%
Unknown	4	0.4%	4	0.5%	0	0.0%
Gender						
Female	613	58.2%	429	57.8%	184	59.0%
Male	441	41.8%	313	42.2%	128	41.0%
Age group, years*						
<40 (< 30 / 30–39)	653	62.0%	486	65.5%	167	53.5%
≧40 (40–49 / 50–59 / ≧60)	401	38.0%	256	34.5%	145	46.5%
Continuous years in pharmacy practice*						
<10 (< 5 / 5–9)	544	51.6%	411	55.4%	133	42.6%
10–19	307	29.1%	207	27.9%	100	32.1%
≧20	203	19.3%	124	16.7%	79	25.3%
Position						
Pharmacist in charge	671	63.7%	465	62.7%	206	66.0%
Staff pharmacist	300	28.5%	216	29.1%	84	26.9%
Temporary or part-time pharmacist	70	6.6%	52	7.0%	18	5.8%
Other	13	1.2%	9	1.2%	4	1.3%
Number of patients receiving medication counseling per day						
<9	55	5.2%	45	6.1%	10	3.2%
10–19	336	31.9%	239	32.2%	97	31.1%
20–29	357	33.9%	251	33.8%	106	34.0%
30–39	227	21.5%	158	21.3%	69	22.1%
≧40	79	7.5%	49	6.6%	30	9.6%
Proportion of patients ≧ 65 years and older receiving medication guidance						
Don’t know	0	0.0%	0	0.0%	0	0.0%
None	8	0.8%	6	0.8%	2	0.6%
10–30%	164	15.6%	118	15.9%	46	14.7%
40–60%	527	50.0%	370	49.9%	157	50.3%
70–90%	350	33.2%	245	33.0%	105	33.7%
All	3	0.3%	1	0.1%	2	0.6%
No answer	2	0.2%	2	0.3%	0	0.0%
Proportion of frail patients ≧ 65 years old receiving medication counseling*						
Don’t know	212	20.1%	189	25.5%	23	7.4%
None	45	4.3%	36	4.9%	9	2.9%
10–30%	579	54.9%	387	52.2%	192	61.5%
40–60%	183	17.4%	111	15.0%	72	23.1%
70–90%	35	3.3%	19	2.6%	16	5.1%
All	0	0.0%	0	0.0%	0	0.0%
Experience in nutritional counseling*						
No (never/rarely)	579	54.9%	478	64.4%	101	32.4%
Yes (frequently/sometimes/occasionally)	475	45.1%	264	35.6%	211	67.6%

Values are presented as number (%) unless otherwise indicated. “Yes” for frailty assessment was defined as any response other than “rarely,” or “never.” (i.e., “occasionally,” “sometimes,” or “always”). Responses were dichotomized for analysis. See Supplemental Table S2 for full response distributions. Asterisk (*) indicates a statistically significant association with frailty assessment (*p* < 0.05, chi-square test or Fisher’s exact test)

**Fig. 1:**
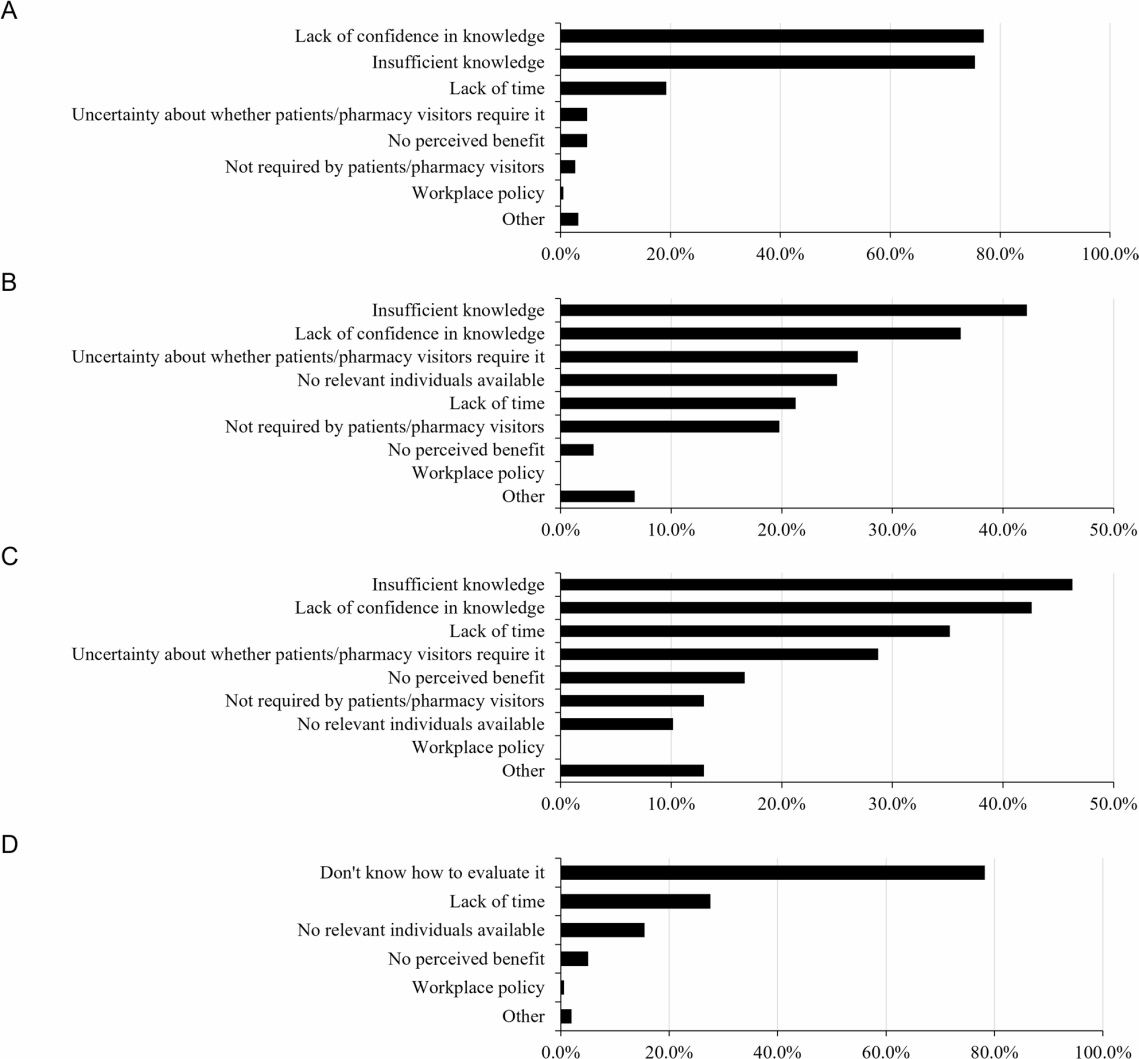
Reasons for pharmacists’ reluctance or inactivity in providing physical activity and exercise support, and for not conducting frailty assessments. The data represent the proportion of respondents selecting each option. Multiple responses were allowed. (**A**) Reasons for community pharmacists feeling reluctant to respond to consultations on physical activity and exercise (*n* = 187). (**B**) Reasons for non-implementation of pharmacist-led support for physical activity and exercise among pharmacists who reported “never” implementing such support (*n* = 268). (**C**) Reasons for community pharmacists holding negative views regarding pharmacist-led support for physical activity and exercise (*n* = 108). (**D**) Reasons for non-assessment of frailty among respondents who answered “never” to having ever conducted a frailty assessment (*n* = 493)

Pharmacists’ involvement in promoting physical activity and exercise is presented in Table 2, with detailed distributions in Supplemental Table S2. More than half of the respondents reported experience responding to consultations regarding physical activity and exercise. Pharmacist-initiated support related to physical activity and exercise was widely reported, and most respondents expressed positive attitudes toward such involvement (Table 2). Insufficient knowledge was identified as a primary reason for resistance to, or failure to implement, physical activity and exercise support (Fig. 1A-C).

**Table 2:** Pharmacists’ perceptions, attitudes and practices on physical activity, exercise and frailty, including educational opportunities

Characteristics			Frailty assessment in pharmacy practice			
	Overall		No		Yes	
	*N* = 1,054	100.0%	*N* = 742	70.4%	*N* = 312	29.6%
Experience in responding to consultations regarding physical activity and exercise*						
No (never/rarely)	782	74.2%	615	82.9%	167	53.5%
Yes (frequently/sometimes/occasionally)	272	25.8%	127	17.1%	145	46.5%
Resistance to responding to consultations regarding physical activity and exercise*						
No (not at all reluctant/not very reluctant/neutral)	867	82.3%	596	80.3%	271	86.9%
Yes (strongly reluctant/somewhat reluctant)	187	17.7%	146	19.7%	41	13.1%
Experience in pharmacist-initiated support for physical activity and exercise*						
No (never/rarely)	616	58.4%	514	69.3%	102	32.7%
Yes (always/sometimes/occasionally)	438	41.6%	228	30.7%	210	67.3%
Intention to engage in pharmacist-initiated support for physical activity and exercise*						
No	108	10.2%	95	12.8%	13	4.2%
Yes	946	89.8%	647	87.2%	299	95.8%
Experience in interprofessional collaboration in supporting physical activity and exercise*						
No (never/rarely)	952	90.3%	697	93.9%	255	81.7%
Yes (frequently/sometimes/occasionally)	102	9.7%	45	6.1%	57	18.3%
Belief: important for pharmacists to *know* patient’s frailty status*						
Disagree/somewhat agree/neither agree or disagree	123	11.7%	99	13.3%	24	7.7%
Strongly agree/agree	931	88.3%	643	86.7%	288	92.3%
Belief: important for pharmacist to *assess* patient’s frailty status*						
Disagree/neither agree or disagree/somewhat agree/don’t know	421	39.9%	352	47.4%	69	22.1%
Strongly agree/agree	633	60.1%	390	52.6%	243	77.9%
Pharmacists support the physical activity and exercise of patients and pharmacy visitors*						
Not necessary at all/Somewhat unnecessary/Neither necessary nor unnecessary	251	23.8%	199	26.8%	52	16.7%
Strongly necessary/somewhat necessary	803	76.2%	543	73.2%	260	83.3%
Pharmacists provide patients and pharmacy visitors with support in nutritional guidance and oral care*						
Not necessary at all/somewhat unnecessary/neither necessary nor unnecessary	203	19.3%	161	21.7%	42	13.5%
Strongly necessary/somewhat necessary	851	80.7%	581	78.3%	270	86.5%
Pharmacists provide opportunities for local residents to engage in mutual interaction*						
Not necessary at all/somewhat unnecessary/neither necessary nor unnecessary	403	38.2%	313	42.2%	90	28.8%
Strongly necessary/somewhat necessary	651	61.8%	429	57.8%	222	71.2%
Recognition of the impact of medications on physical activity*						
No/I don’t know	116	11.0%	94	12.7%	22	7.1%
Yes	938	89.0%	648	87.3%	290	92.9%
Recognition of the concept of “rehabilitation pharmacotherapy”*						
I don’t know/I haven’t heard	910	86.3%	679	91.5%	231	74.0%
I know well/I know	144	13.7%	63	8.5%	81	26.0%
Educational opportunities related to supporting physical activity and exercise*						
Never	832	78.9%	626	84.4%	206	66.0%
Several times a year/several times in the past/completed a series	220	20.9%	114	15.4%	106	34.0%
No answer	2	0.2%	2	0.3%	0	0.0%
Willingness to learn about supporting physical activity and exercise*						
Not interested	53	5.0%	45	6.1%	8	2.6%
If it became necessary	318	30.2%	260	35.0%	58	18.6%
If the opportunity arises	595	56.5%	393	53.0%	202	64.7%
Highly motivated	88	8.3%	44	5.9%	44	14.1%
Willingness to learn about “rehabilitation pharmacotherapy”*						
Not interested	44	4.2%	37	5.0%	7	2.2%
If it became necessary	282	26.8%	225	30.3%	57	18.3%
If the opportunity arises	600	56.9%	406	54.7%	194	62.2%
Highly motivated	128	12.1%	74	10.0%	54	17.3%

Values are presented as number (%) unless otherwise indicated. “Yes” for frailty assessment was defined as any response other than “rarely,” or “never.” (i.e., “occasionally,” “sometimes,” or “always”). Responses were dichotomized for analysis. See Supplemental Table S2 for full response distributions. Asterisk (*) indicates a statistically significant association with frailty assessment (*p* < 0.05, chi-square test or Fisher’s exact test)

**Fig. 2:**
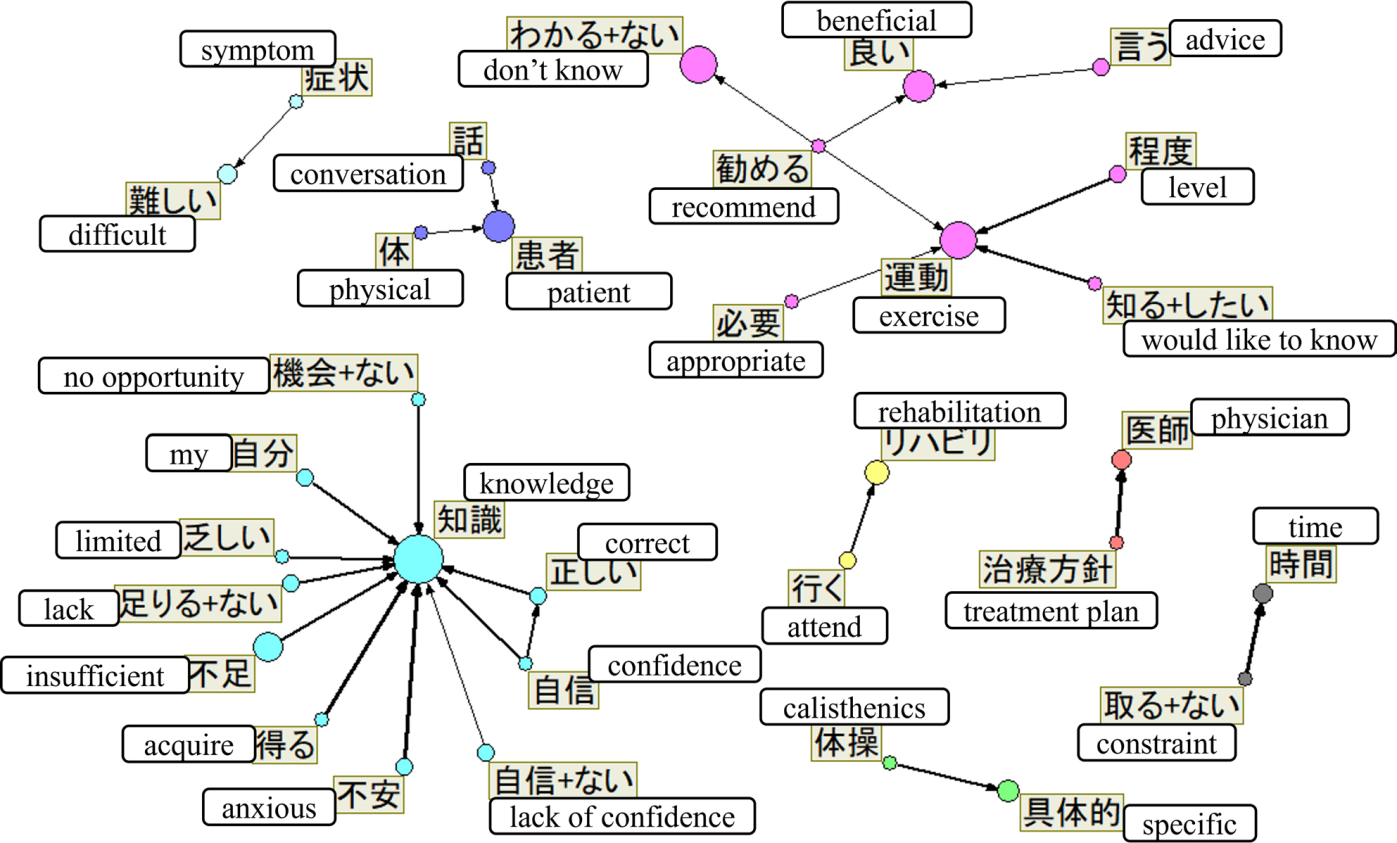
Co-occurrence networks of free-text responses regarding pharmacist’ concerns about physical activity support. The data shows the co-occurrence network of descriptive responses to the question. Regarding pharmacists’ concerns about providing physical activity and exercise support, expressions such as “*I’m not confident in my knowledge*,” “*I feel anxious*,” “*I lack knowledge*,” and “*I do not know what level of exercise is appropriate for the patient.*” were identified, indicating worries about their own expertise

The text-mining analysis of the free-text responses revealed pharmacists’ concerns regarding physical activity and exercise support included lack of knowledge, as well as lack of confidence and anxiety related to their knowledge (Fig. 2). In addition, responses such as not knowing what types of exercise to recommend and difficulty securing time were also identified. Additional results of the free-text analysis are presented in Supplemental Figure S1.

Pharmacists’ awareness, attitudes, and practices concerning frailty are presented in Table 2, with detailed distributions in Supplemental Table S2. A total of 88.3% and 60.1% of the pharmacists agreed that it was important to know and assess the frailty status of patients, respectively (Table 2). Notably, only 29.6% of pharmacists reported conducting frailty assessments in their practice (Table 2). Among pharmacists who had never conducted frailty assessments, the most frequently reported reason for non-implementation was “Don’t know how to evaluate it” (Fig. 1D). Pharmacists who reported conducting frailty assessments were more likely to be aged 40 years or older, have longer professional experience, and have prior experience in nutritional counseling (Table 1). In addition, they reported greater involvement in consultations related to physical activity and exercise, greater engagement in pharmacist-initiated support, more positive attitudes toward such activities, and more experience with interprofessional collaboration (Table 2).

**Fig. 3:**
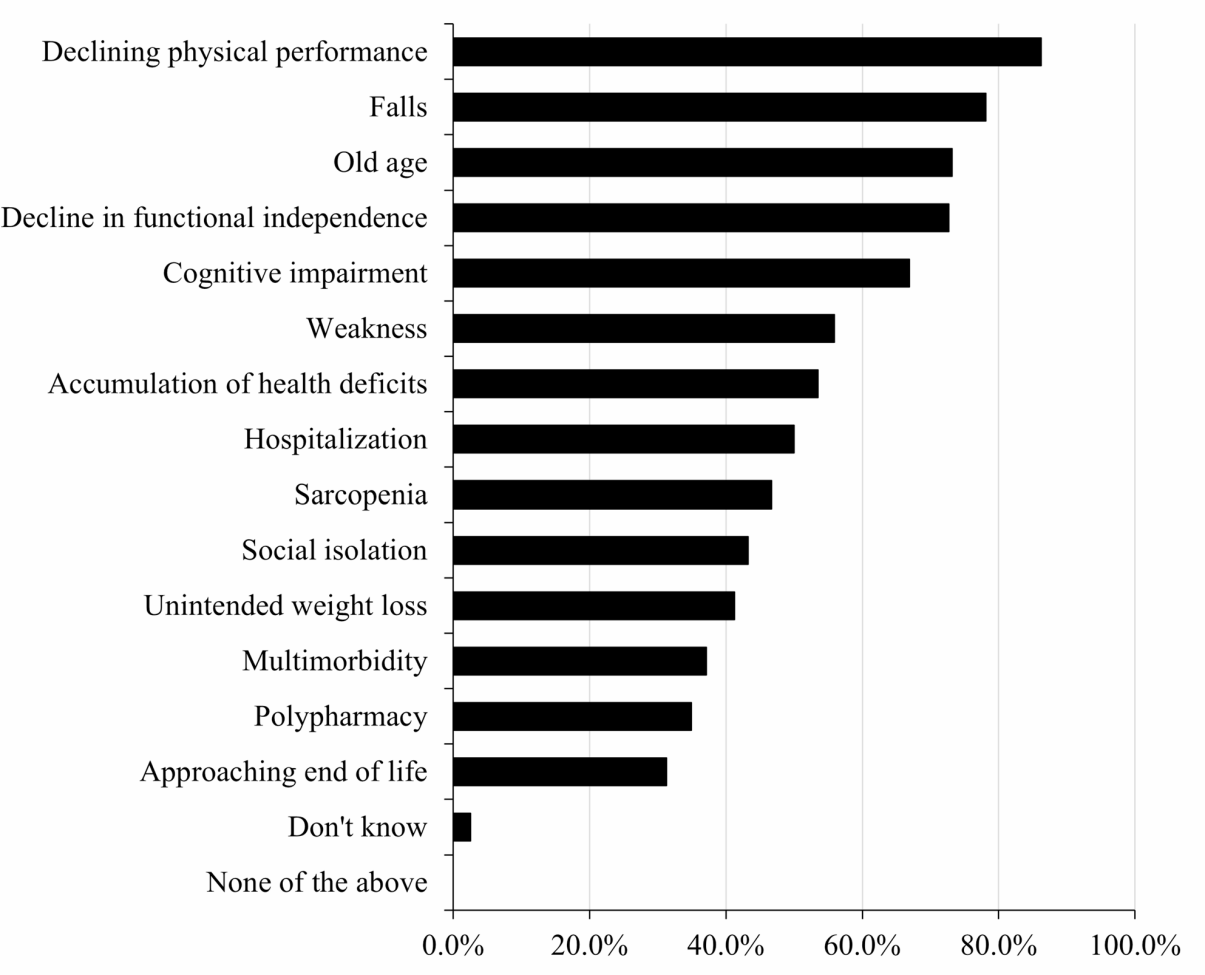
Health items considered to be associated with frailty. The data represents the percentage of respondents (*n* = 1,054) who considered each item to be associated with frailty. Multiple responses were allowed

Over 70% of respondents associated frailty with health-related factors linked to physical function, such as decline in physical performance, falls, old age, and decline in functional independence (Fig. 3). In contrast, less than half of the respondents recognized other relevant health aspects (e.g., multimorbidity, polypharmacy, approaching end of life, unintended weight loss, and social isolation) as being related to frailty (Fig. 3). Over 60% of pharmacists agreed that they should be involved in frailty-related care, including physical activity, nutrition and oral care, and promoting social interaction (Table 2). Although most respondents reported recognizing the influence of medications on physical activity, only a small proportion were familiar with the concept of rehabilitation pharmacotherapy (Table 2). Furthermore, while relatively few pharmacists reported having received formal educational opportunities related to physical activity and exercise support, a large majority expressed interest in pursuing such education in the future (Table 2).

The associations between respondent characteristics and implementation of the frailty assessment are presented in Table 3. Multivariate logistic regression analysis identified several factors independently associated with the implementation of frailty assessment in pharmacy practice. Pharmacists who were engaged in consultations related to physical activity and exercise, and provided pharmacist-initiated support for physical activity were more likely to conduct frailty assessments. In addition, recognition of the importance of frailty assessment, familiarity with rehabilitation pharmacotherapy, and prior educational experience related to physical activity and exercise support were also significantly associated with frailty assessment practices. Similar results were observed in a sensitivity analysis (Supplemental Table S4).

**Table 3:** Associations between respondent characteristics and assessment of frailty in pharmacy practice

Characteristics	Crude odds ratio (95% CI)	Adjusted odds ratio (95% CI)
Age group, years (ref = < 40)		
≧40	1.64 (1.25–2.15)	2.20 (1.59–3.04)*
Experience in nutritional counseling (ref = rarely or never)		
Frequently/sometimes/occasionally	3.78 (2.85–5.00)	1.83 (1.31–2.56)*
Experience in responding to consultations regarding physical activity and exercise (ref = rarely or never)		
Frequently/sometimes/occasionally	4.20 (3.13–5.63)	1.73 (1.19–2.51)*
Experience in pharmacist-initiated support for physical activity and exercise (ref = rarely or never)		
Always/sometimes/occasionally	4.64 (3.49–6.16)	2.46 (1.73–3.50)*
Experience in interprofessional collaboration in supporting physical activity and exercise (ref = rarely or never)		
Frequently/sometimes/occasionally	3.46 (2.28–5.25)	1.58 (0.95–2.60)
Belief: important for pharmacist to *assess* patient’s frailty status		
Disagree/neither agree or disagree/somewhat agree/don’t know	1	1
Strong agree/agree	3.17 (2.34–4.30)	2.83 (1.97–4.07)*
Pharmacists support the physical activity and exercise of patients and pharmacy visitors		
Not necessary at all/somewhat unnecessary/neither necessary nor unnecessary	1	1
Strongly necessary/somewhat necessary	1.83 (1.30–2.57)	0.91 (0.56–1.47)
Pharmacists provide patients and pharmacy visitors with support in nutritional guidance and oral care		
Not necessary at all/somewhat unnecessary/neither necessary nor unnecessary	1	1
Strongly necessary/somewhat necessary	1.78 (1.23–2.57)	1.07 (0.64–1.77)
Pharmacists provide opportunities for local residents to engage in mutual interactions		
Disagree/neither agree or disagree/somewhat agree/don’t know	1	1
Strongly agree/agree	1.79 (1.35–2.39)	1.07 (0.74–1.54)
Recognition of the concept of “rehabilitation pharmacotherapy”		
I don’t know/I haven’t heard	1	1
I know well/I know	3.77 (2.63–5.42)	3.05 (1.99–4.67)*
Educational opportunities related to supporting physical activity and exercise (ref = never)		
Several times a year/several times in the past/completed a series	2.82 (2.07–3.84)	1.62 (1.12–2.34)*
Willingness to learn about supporting physical activity and exercise (ref = not interested)		
Highly motivated/if the opportunity arises/if it became necessary	2.45 (1.14–5.26)	0.74 (0.21–2.64)
Willingness to learn about “rehabilitation pharmacotherapy” (ref = not interested)		
Highly motivated/if the opportunity arises/if it became necessary	2.28 (1.00–5.18)	0.97 (0.24–3.82)

Crude odds ratios and adjusted odds ratios (AORs) with 95% confidence intervals are presented. An odds ratio greater than 1 indicates a higher likelihood of conducting frailty assessment (Yes: “always,” “sometimes,” or “occasionally”; No: “rarely,” or “never”) associated with the corresponding factor. AORs were estimated using multivariate logistic regression that included only variables found to be statistically significant (*p* < 0.05) in univariate analysis. Statistically significant AORs (*p* < 0.05) are marked with an asterisk (*). Reference categories are indicated in parentheses

## Discussion

This study investigated the perceptions, attitudes, and practices of community pharmacists in Japan regarding physical activity, exercise support, and frailty. The most significant finding was that, although many pharmacists recognized the importance of supporting physical activity and exercise and expressed proactive attitudes, lack of knowledge remained a major barrier to implementation. Another key finding was that despite widespread recognition of the importance of frailty, the actual rate of frailty assessments practice remained low. Multivariate logistic regression analysis showed that active involvement in physical activity support, positive recognition of frailty interventions, educational experience related to physical activity, and familiarity with rehabilitation pharmacotherapy were independently associated with the implementation of frailty assessment. These findings suggest that promoting frailty assessments in community pharmacy practice requires pharmacists’ active involvement in physical activity support, a positive recognition of the significance of frailty interventions, and access to relevant educational opportunities.

This study demonstrated that many community pharmacists recognize the importance of promoting physical activity and exercise as strategies to prevent or mitigate frailty. Although they generally expressed positive attitudes toward providing such support, insufficient knowledge was frequently identified as a major barrier to practical implementation. As shown in Table 2, most pharmacists considered physical activity and exercise support necessary for frailty prevention. However, free-text responses such as “*I am concerned about whether I am conveying accurate information*” or “*I do not have sufficient knowledge*,* so I cannot provide advice with confidence*,” reflected anxiety and a lack of confidence related to insufficient knowledge, suggesting that low self-efficacy regarding knowledge may hinder practical support efforts (Fig. 2). In Japan, approximately 40% of pharmacists reportedly feel unable to provide adequate advice during health consultations owing to insufficient knowledge [23]. Given that physical activity and exercise are regarded as key components of health consultations in pharmacies [24], acquisition of the requisite knowledge for providing such support is essential. In addition, analysis of the free-text responses suggested that pharmacists experienced psychological barriers that were not solely related to insufficient knowledge. Comments such as “*I am not confident that I can provide advice appropriately tailored to a patient’s physical condition*” and “*I worry that recommending exercise might lead to other problems*,* such as worsening back pain*” indicate that anxiety and lack of confidence may independently hinder pharmacists’ engagement in physical activity support. These findings suggest that the issue extends beyond a simple shortage of information, and that low self-efficacy in applying knowledge in practice may suppress pharmacists’ engagement in such support. Therefore, educational interventions for pharmacists should focus not only on providing knowledge but also on strengthening practical counseling skills and confidence in delivering guidance.

Nevertheless, the finding that lack of knowledge was the primary barrier should be interpreted cautiously. In questionnaire-based surveys, respondents may select lack of knowledge as a socially acceptable explanation for difficulties in implementing new practices. Therefore, the response may not fully reflect the true underlying barriers. Pharmacists in Japan perform a wide range of duties, including dispensing, medication counseling and health promotion, which often involve considerable time and physical workload. Accordingly, as observed in this study, time constraints may also represent a major barrier (Fig. 1). Frailty assessment tools vary from comprehensive assessments requiring substantial time to brief screening tools that can be easily incorporated into routine practice [25, 26]. Increasing awareness of practical assessment tools suitable for busy pharmacy settings may facilitate behavioral changes toward frailty prevention.

In this study, none of the respondents identified workplace policy regarding physical activity support or frailty prevention as a factor. However, because the survey was conducted through cooperating companies, respondents may have hesitated to select such responses due to concerns that their answers could be identifiable by their employers. Future studies should therefore examine the influence of factors other than knowledge on pharmacists’ behaviors related to frailty prevention.

A significant association was observed between physical activity support and frailty assessment, suggesting that pharmacists already engaged in preventive activities may be more likely to conduct frailty assessments as part of their routine practice. Pharmacists who actively provide advice on exercise may also pay greater attention to age-related functional decline, thereby increasing their likelihood of assessing frailty. This finding indicates that promoting engagement in physical activity support may indirectly facilitate the implementation of frailty assessment in community pharmacy settings.

In contrast, although many pharmacists understood the importance of recognizing frailty, the proportion of pharmacists who had experienced conducting frailty assessments was relatively low (Table 2). Only 11.1% of pharmacists reported always or sometimes conducting frailty assessments, suggesting that frailty assessment is not routinely implemented in community pharmacy practice (Supplemental Table S2). In Japan, pharmacists are authorized to engage in dispensing, medication counseling, and health promotion but are not permitted to perform medical practices such as diagnosis or prescribing. Consequently, pharmacists may perceive frailty assessment as involving diagnostic elements and may therefore hesitate to perform it because of professional role boundaries [2, 21, 25, 26]. Previous studies have also reported limited knowledge of frailty assessment and underutilization of available assessment tools among pharmacists [27], suggesting that an insufficient understanding of assessment methods may further impede the implementation of frailty assessments in community pharmacy settings.

Age was independently associated with the implementation of frailty assessment in the multivariate analysis. In Japan, pharmacy education transitioned from a four-year to a six-year program in 2006, with enhanced practical training; however, frailty-related content has not yet been fully integrated into the curriculum [28]. As a result, knowledge acquisition likely depends on postgraduate education, and older pharmacists may have had greater exposure to such training. In addition, with increasing experience, pharmacists are more likely to encounter older patients and frailty-related clinical changes, which may facilitate recognition of frailty as a routine clinical issue and promote its assessment. Furthermore, in the nursing field [29], accumulated clinical experience has been associated with improved communication skills and the ability to identify patient needs. Similarly, among pharmacists, increasing years of experience may enhance the ability to identify and respond to patient needs. Together with the development of patient trust, this may encourage patients to disclose changes in their condition and thereby facilitate the initiation of intervention-related actions, such as frailty assessment, even when knowledge is not fully sufficient. Taken together, promoting frailty assessment may require not only knowledge-based education but also opportunities to gain practical experience and the development of patient trust.

Frailty prevention requires a balanced approach that includes physical activity, nutrition (diet and oral function), and social participation [30, 31]. In recent years, oral frailty, characterized by declines in oral function, has been associated with an increased risk of future frailty and mortality, highlighting the importance of comprehensive support including oral care and nutritional guidance [32]. In this study, approximately 80% of respondents recognized the importance of these aspects (Table 2), and experience with nutritional counseling was significantly associated with the implementation of frailty assessment (Table 3). This association may reflect the fact that pharmacists engaged in nutritional counseling are more likely to assess multiple aspects of patients’ health, including nutritional status and oral function, thereby enhancing their understanding of frailty. In addition, such engagement may reflect a shift from a disease-centered to a more patient- or life-centered care approach, which may facilitate recognition of frailty as a clinically relevant issue and promote its assessment. Further research is needed to clarify the role of pharmacists in nutritional counseling and oral frailty management.

Structured educational opportunities for community pharmacists appear essential for promoting frailty assessment. Pharmacists who had received education on physical activity and exercise support were more likely to conduct frailty assessments (Table 2). Free-text responses also highlighted the need for improved knowledge and instructional skills tailored to patient conditions, such as “*frailty-preventive exercises that can be performed at home*” and “*exercise methods adjusted to physical ability*” (Supplemental Figure S1C). Effective frailty management requires a comprehensive understanding of physical, psychological, and social factors [3, 30, 31, 33]. Consistent with previous study [21], relatively few pharmacists in this study identified social and psychological factors as being related to frailty (Fig. 3), suggesting that pharmacists’ understanding of frailty remains limited and fragmented. Awareness of polypharmacy as a frailty-related issue was also limited (Fig. 3). Given their expertise in pharmacotherapy, this finding underscores the importance of explicitly educating pharmacists on the intersection between frailty and medication management. Additionally, although only a small proportion of the respondents were familiar with the concept of rehabilitation pharmacotherapy, this recognition was significantly associated with the implementation of frailty assessment (Table 3). Rehabilitation pharmacotherapy aims to optimize medication management to maintain or improve physical function and facilitate rehabilitation while avoiding adverse events that may interfere with rehabilitation [34]. Because this approach emphasizes functional status, pharmacists who recognize this concept may be more aware of the importance of assessing physical function and therefore more likely to attempt frailty assessment in practice. This study also confirmed that some pharmacists experience difficulties regarding the relationship between physical activity or exercise support and rehabilitation (Fig. 2). Promoting understanding of rehabilitation pharmacotherapy may therefore encourage behavioral changes in pharmacists’ involvement in physical activity support and frailty prevention. Moving forward, structured educational programs that promote a holistic understanding of frailty while incorporating the concepts of polypharmacy and rehabilitation pharmacotherapy need to be developed and evaluated for community pharmacists.

The major strength of this study is its comprehensive elucidation of community pharmacists’ perceptions, attitudes, and practices regarding support for physical activity, exercise, and frailty management. Few such studies have been conducted in Japan, making this investigation novel. In particular, this study had three strengths. First, by conducting a nationwide survey rather than limiting the sample to a specific region, we obtained data with high generalizability. Second, we comprehensively clarified not only the extent to which community pharmacists are involved in physical activity support and frailty management but also their perceptions and awareness regarding these roles. Finally, specific barriers that hinder the implementation of physical activity support and frailty assessment in practice were identified. This clearly demonstrated the need for educational support to overcome these barriers. These findings offer valuable insights into the role of pharmacists in supporting older adults in community settings with important implications for both practice and education.

However, several limitations should be considered. The relatively low response rate in this study may be explained by several factors. First, the questionnaire contained a large number of items, which may have increased respondent burden and led to survey discontinuation. Second, although the survey was conducted anonymously, it was disseminated through cooperation companies; therefore, some pharmacists may have hesitated to participate due to concerns that their responses or access logs could potentially be identifiable by their employer. Finally, the survey was conducted during a particularly busy period from the year-end through the end of the fiscal year, which may have further limited participation. Consequently, pharmacists with greater interest in frailty prevention and physical activity support may have been more likely to respond, potentially leading to an overestimation of pharmacists’ engagement in these activities.

Compared with national government statistics (Supplemental Table S3), some differences were observed in the demographic characteristics of respondents, although the overall patterns were broadly comparable. In particular, younger pharmacists were relatively overrepresented in the study population, suggesting that the sample may reflect a comparatively less experienced cohort of pharmacists in Japan. Younger pharmacists may be more receptive to emerging concepts and preventive approaches while having less confidence in conducting structured clinical assessments because of limited professional experience. This age-related imbalance may partly explain the discrepancy observed in this study between positive attitudes toward physical activity support and the relatively low implementation of frailty assessment. Therefore, caution is warranted when generalizing these findings to the broader population of community pharmacists in Japan.

In this study, frailty assessment was not defined using any specific standardized instrument. Therefore, respondents may have interpreted frailty assessments differently, ranging from the use of validated assessment tools to judgments based on informal clinical impressions. The observed association between frailty assessment, physical activity support, and educational opportunities may therefore reflect pharmacists’ subjective judgments regarding patients’ frailty status rather than the use of standardized assessments. Consequently, not all pharmacists who reported conducting frailty assessments were necessarily implementing clinically meaningful or standardized evaluation. Future investigation should examine pharmacists’ knowledge and implementation of specific frailty assessment methods in greater detail.

This study has several additional limitations. Because the participating organizations were selected by the researchers, which may have introduced a selection bias owing to the characteristics or policies specific to the selected companies. In particular, the participants were employed by companies that were actively involved in community-based healthcare activities. They may have received education and support from their organizations regarding physical activity support and frailty prevention. Therefore, such organizational support may have influenced their responses, potentially leading to an overestimation of pharmacists’ engagement in physical activity support and frailty-related practices. Second, pharmacists with a pre-existing interest in physical activity support or frailty prevention may have been more likely to participate in the survey. Consequently, pharmacists who are more motivated or actively engaged in such activities may be overrepresented in the sample, potentially leading to an overestimation of the overall level of awareness and engagement among community pharmacists. Third, the participants may have overestimated their responses to some questionnaire items.

Despite these limitations, this study provides important implications for pharmacy practice and education. In the current Japanese pharmacy education curriculum [28], topics related to frailty and physical activity are not systematically incorporated, and standardization of such education has not been established. As a result, opportunities for pharmacists to acquire knowledge of them remain limited, which may contribute to insufficient knowledge in clinical practice. Previous studies have reported that educational interventions incorporating blended learning and active learning for healthcare professionals are effective in improving their knowledge, attitudes, and behaviors [35]. To promote the implementation of frailty prevention, including assessment, it is crucial to develop educational programs that combine multiple educational methods, such as lectures and group work, covering topics such as methods of supporting physical activity and exercise, interprofessional collaboration, the definition and assessment of frailty and their significance, as well as the relationship between physical activity and pharmacotherapy, including rehabilitation therapy. The effectiveness of such programs should then be evaluated in terms of its impact on behavioral change among community pharmacists.

## Conclusion

This study revealed that although many community pharmacists recognize the importance of supporting physical activity, exercise, and frailty management, insufficient knowledge remains a practical barrier to their active engagement in these areas. Consequently, the implementation rate of frailty assessment in community pharmacy practice remains low. To promote the implementation of frailty prevention, including assessment, it is crucial to enhance educational opportunities and encourage active pharmacist involvement in supporting physical activity and developing a positive perception of frailty assessments. These findings suggest that incorporating education in physical assessment and frailty care into community pharmacist training may enhance future engagement in frailty prevention.

## Supplementary Information

Below is the link to the electronic supplementary material.

Supplementary Material 1

## Data Availability

All data generated or analyzed during this study are included in this published article.
