# Supplementary material for: A survey of community pharmacists’ perceptions, attitudes, and practices regarding support for physical activity, exercise, and frailty
Source: J Pharm Health Care Sci. 2026 May 28;12:66. doi: 10.1186/s40780-026-00588-w (PMC13397794; doi:10.1186/s40780-026-00588-w)
Supplement: Supplementary file 1 — Supplementary Material 1 [file 40780_2026_588_MOESM1_ESM.docx]

**Supplemental Tables**

**Table S1. Survey items**

| No. | Question | Statement | Answer format | Answer options | Categories |
| --- | --- | --- | --- | --- | --- |
| 1 | Please indicate the prefecture in which the pharmacy you are currently working at is located. |  | Single | Hokkaido | Hokkaido |
|  |  |  |  | Aomori/Iwate/Miyagi/Akita/Yamagata/Fukushima | Tohoku |
|  |  |  |  | Ibaraki/Tochigi/Gunma/Saitama/Chiba/Tokyo/Kanagawa | Kanto |
|  |  |  |  | Niigata/Toyama/Ishikawa/Fukui/Yamanashi/Nagano/Gifu/Shizuoka/Aichi | Chubu |
|  |  |  |  | Mie/Shiga/Kyoto/Osaka/Hyogo/Nara/Wakayama | Kinki |
|  |  |  |  | Tottori/Shimane/Okayama/Hiroshima/Yamaguchi/Tokushima/Kagawa/Ehime/Kochi | Chugoku-Shikoku |
|  |  |  |  | Fukuoka/Saga/Nagasaki/Kumamoto/Oita/Miyazaki/Kagoshima/Okinawa | Kyusyu |
| 2 | Please indicate the location setting of the pharmacy where you are currently employed. |  | Single | Pharmacy adjacent to a large hospital (400 beds or more) | Point-based dispensing pharmacies |
|  |  |  |  | Pharmacy adjacent to a small or medium-sized hospital (less than 400 beds) |  |
|  |  |  |  | Pharmacy located next to a clinic or medical office |  |
|  |  |  |  | Pharmacy located near (but not adjacent to) a clinic |  |
|  |  |  |  | Community-based pharmacy serving a wide range of prescriptions (not adjacent to a specific medical institution) | Area-based dispensing pharmacies |
|  |  |  |  | Pharmacy serving multiple medical institutions within a medical mall or similar complex |  |
|  |  |  |  | Pharmacy attached to a drugstore | Pharmacy-based drugstores |
| 3 | Please indicate the number of full-time equivalent pharmacists working per day at your current pharmacy. |  | Single | 1 | <2 |
|  |  |  |  | 1.1–2 |  |
|  |  |  |  | 2.1–3 | 2–4 |
|  |  |  |  | 3.1–4 |  |
|  |  |  |  | 4.1–5 | 4> |
|  |  |  |  | >5–1 |  |
|  |  |  |  | Unknown | Unknown |
| 4 | Please indicate our gender. |  | Single | Female |  |
|  |  |  |  | Male |  |
| 5 | Please indicate our age. |  | Single | <30 | <40 |
|  |  |  |  | 30–39 |  |
|  |  |  |  | 40–49 | >40 |
|  |  |  |  | 50–59 |  |
|  |  |  |  | >60 |  |
| 6 | How many years have you been working as a community pharmacist? |  | Single | <5 | <10 |
|  |  |  |  | 5–9 |  |
|  |  |  |  | 10–19 | 10–19 |
|  |  |  |  | >20 | >20 |
| 7 | Please indicate your position at the pharmacy. |  | Single | Pharmacist in charge |  |
|  |  |  |  | Staff pharmacist |  |
|  |  |  |  | Temporary or part-time pharmacist |  |
|  |  |  |  | Other |  |
| 8 | On average, how many patients do you provide medication counseling to per day? |  | Single | <9 |  |
|  |  |  |  | 10–19 |  |
|  |  |  |  | 20–29 |  |
|  |  |  |  | 30–39 |  |
|  |  |  |  | >40 |  |
| 9 | Among the patients for whom you provide medication counseling, what is the number of patients >65 years old? |  | Single | Don’t know |  |
|  |  |  |  | None |  |
|  |  |  |  | 10–30% |  |
|  |  |  |  | 40–60% |  |
|  |  |  |  | 70–90% |  |
|  |  |  |  | All |  |
| 10 | How many patients >65 years old receiving medication counseling at the pharmacy do you consider to be in frailty? |  | Single | Don’t know |  |
|  |  |  |  | None |  |
|  |  |  |  | 10–30% |  |
|  |  |  |  | 40–60% |  |
|  |  |  |  | 70–90% |  |
|  |  |  |  | All |  |
| 11 | Please describe your experience in providing nutritional counseling in your pharmacy practice. |  | Single | Never | No |
|  |  |  |  | Rarely | Yes |
|  |  |  |  | Occasionally |  |
|  |  |  |  | Sometimes |  |
|  |  |  |  | Frequently |  |
| 12 | How often do you respond to consultations from patients/visitors regarding physical activity or exercise (including rehabilitation)? |  | Single | Never | No |
|  |  |  |  | Rarely | Yes |
|  |  |  |  | Occasionally |  |
|  |  |  |  | Sometimes |  |
|  |  |  |  | Frequently |  |
| 13 | Question for those who answered “Yes” to Q12. Please list the specific consultations you have received from patients/visitors regarding physical activity or exercise (including rehabilitation) in bullet points, to the extent possible. |  | Free text |  |  |
| 14 | Do you feel any reluctance to responding to consultations from patients or pharmacy/visitors regarding physical activity or exercise (including rehabilitation)? |  | Single | Not at all reluctant | No |
|  |  |  |  | Not very reluctant |  |
|  |  |  |  | Neutral |  |
|  |  |  |  | Somewhat reluctant | Yes |
|  |  |  |  | Strongly reluctant |  |
| 15 | Question for those who answered “Yes” to Q14. Please explain the reasons for your response. |  | Multiple | Lack of time |  |
|  |  |  |  | Lack of confidence in knowledge |  |
|  |  |  |  | Insufficient knowledge |  |
|  |  |  |  | No perceived benefit |  |
|  |  |  |  | Workplace policy |  |
|  |  |  |  | Not required by patients/pharmacy visitors |  |
|  |  |  |  | Uncertainty about whether patients/pharmacy visitors require it |  |
|  |  |  |  | Other |  |
| 16 | How frequently do you actively provide pharmacists-led suggestions or advice to patients/visitors regarding physical activity or exercise (including rehabilitation)? |  | Single | Never | No |
|  |  |  |  | Rarely | Yes |
|  |  |  |  | Occasionally |  |
|  |  |  |  | Sometimes |  |
|  |  |  |  | Always |  |
| 17 | Question for those who answered “Never” to Q16. Please indicate the reasons why you do not provide suggestions or advice regarding physical activity or exercise (including rehabilitation) to patients/visitors. |  | Multiple | Lack of time |  |
|  |  |  |  | Lack of confidence in knowledge |  |
|  |  |  |  | Insufficient knowledge |  |
|  |  |  |  | No perceived benefit |  |
|  |  |  |  | Workplace policy |  |
|  |  |  |  | No relevant individuals available |  |
|  |  |  |  | Not required by patients/pharmacy visitors |  |
|  |  |  |  | Uncertainty about whether patients/pharmacy visitors require it |  |
|  |  |  |  | Other |  |
| 18 | Question for those who answered “Sometimes,” “Occasionally,” or “Rarely” to Q16. Please indicate the reasons why you did not provide suggestions or advice regarding physical activity/exercise (including rehabilitation) to patients/visitors. |  | Multiple | Lack of time |  |
|  |  |  |  | Lack of confidence in knowledge |  |
|  |  |  |  | Insufficient knowledge |  |
|  |  |  |  | No perceived benefit |  |
|  |  |  |  | Workplace policy |  |
|  |  |  |  | No relevant individuals available |  |
|  |  |  |  | Not required by patients/pharmacy visitors |  |
|  |  |  |  | Uncertainty about whether patients/pharmacy visitors require it |  |
|  |  |  |  | Other | - |
| 19 | Question for those who answered “Yes” to Q16. Please list the contents of any suggestions or advice related to physical activity/exercise (including rehabilitation) that you have provided or recommended in bullet points, to the extent possible. |  | Free text |  | - |
| 20 | In the future, do you intend to (or continue to) provide patients/visitors with pharmacists-led suggestions or advice regarding physical activity and exercise (including rehabilitation)? |  | Single | No | - |
|  |  |  |  | Yes |  |
| 21 | Question for those who answered “No” to Q20. Please explain the reasons for your response. |  | Multiple | Lack of time |  |
|  |  |  |  | Lack of confidence in knowledge |  |
|  |  |  |  | Insufficient knowledge |  |
|  |  |  |  | No perceived benefit |  |
|  |  |  |  | Workplace policy |  |
|  |  |  |  | No relevant individuals available |  |
|  |  |  |  | Not required by patients/pharmacy visitors |  |
|  |  |  |  | Uncertainty about whether patients/pharmacy visitors require it |  |
|  |  |  |  | Other |  |
| 22 | Do you collaborate with other healthcare professionals (e.g., through referrals or support requests) when responding to consultations or providing suggestions/advice regarding physical activity or exercise (including rehabilitation)? |  | Single | Never | No |
|  |  |  |  | Rarely | Yes |
|  |  |  |  | Occasionally |  |
|  |  |  |  | Sometimes |  |
|  |  |  |  | Frequently |  |
| 23 | Please freely describe any difficulties or challenges you have encountered or are currently encountering when providing consultations, suggestions, or advice regarding physical activity and exercise (including rehabilitation) to patients or visitors. |  | Free text |  |  |
| 24 | Please share your thoughts on the following statement. | It is important for a pharmacist to  *know* a patient’s frailty status. | Single | Disagree | Negative |
|  |  |  |  | Somewhat agree |  |
|  |  | It is important for a pharmacist to  *assess* a patient’s frailty status. |  | Neither agree or disagree |  |
|  |  |  |  | Agree | Positive |
|  |  |  |  | Strongly agree |  |
| 25 | Do you assess whether patients or visitors to your pharmacy are frail as part of your routine practice? |  | Single | Never | No |
|  |  |  |  | Rarely | Yes |
|  |  |  |  | Occasionally |  |
|  |  |  |  | Sometimes |  |
|  |  |  |  | Always |  |
| 26 | Question for those who answered “No” to Q25. Please explain the reasons for your response. |  | Multiple | Lack of time |  |
|  |  |  |  | Don’t know how to evaluate it |  |
|  |  |  |  | No perceived benefit |  |
|  |  |  |  | Workplace policy |  |
|  |  |  |  | No relevant individuals available |  |
|  |  |  |  | Other |  |
| 27 | Considering the list below, please select item(s) that you believe is/are related to frailty. |  | Multiple | Accumulation of health deficits |  |
|  |  |  |  | Approaching end of life |  |
|  |  |  |  | Cognitive impairment |  |
|  |  |  |  | Decline in functional independence |  |
|  |  |  |  | Declining physical performance |  |
|  |  |  |  | Falls |  |
|  |  |  |  | Hospitalization |  |
|  |  |  |  | Multimorbidity |  |
|  |  |  |  | Old age |  |
|  |  |  |  | Polypharmacy |  |
|  |  |  |  | Social isolation |  |
|  |  |  |  | Unintended weight loss |  |
|  |  |  |  | Weakness |  |
|  |  |  |  | Sarcopenia |  |
|  |  |  |  | Don’t know |  |
|  |  |  |  | None of the above |  |
| 28 | Do you think the following items are necessary for pharmacists working in community pharmacies? Please share your opinion. | Pharmacists support the physical activity and exercise of patients and pharmacy visitors | Single | Not necessary at all | Negative |
|  |  |  |  | Somewhat unnecessary |  |
|  |  | Pharmacists provide patients and pharmacy visitors with support in nutritional consultation and oral care |  | Neither necessary nor unnecessary |  |
|  |  | Pharmacists provide opportunities for local residents to engage in mutual interaction |  | Somewhat necessary | Positive |
|  |  |  |  | Strongly necessary |  |
| 29 | Do you think that medications can affect physical activity or exercise (including rehabilitation)? |  | Single | I don’t know |  |
|  |  |  |  | No |  |
|  |  |  |  | Yes |  |
| 30 | Do you know the meaning of the concept of “rehabilitation pharmacotherapy”? |  | Single | I haven’t heard | No |
|  |  |  |  | I don’t know |  |
|  |  |  |  | I know | Yes |
|  |  |  |  | I know well |  |
| 31 | Have you ever received education or training related to supporting physical activity and exercise (including rehabilitation), such as responding to consultations or making recommendations? |  | Single | Never | No |
|  |  |  |  | Completed a series | Yes |
|  |  |  |  | Several times in the past |  |
|  |  |  |  | Several times a year |  |
| 32 | Would you like to learn more about supporting physical activity and exercise (including rehabilitation) in the future, such as responding to consultations or making recommendations? |  | Single | Not interested | Negative |
|  |  |  |  | If it became necessary | Positive |
|  |  |  |  | If the opportunity arises |  |
|  |  |  |  | Highly motivated |  |
| 33 | Would you like to learn more about “rehabilitation pharmacotherapy” in the future? |  | Single | Not interested | Negative |
|  |  |  |  | If it became necessary | Positive |
|  |  |  |  | If the opportunity arises |  |
|  |  |  |  | Highly motivated |  |

**Table S2. Frequency distributions of categorized responses for questionnaire items included in Tables 1–2.**

|  |  |  | Frailty assessment in pharmacy practice | | | | | | | | |
| --- | --- | --- | --- | --- | --- | --- | --- | --- | --- | --- | --- |
|  | Overall |  | Never |  | Rarely |  | Sometimes |  | Often |  | Always |
| Characteristics | N = 1,054 | | N = 493 | | N = 249 | | N = 194 | | N = 99 | | N = 19 |
| Pharmacy practice models | | | | | | | | | | | |
| Pharmacy adjacent to a large hospital (400 beds or more) | 51 |  | 26 |  | 11 |  | 11 |  | 3 |  | 0 |
| Pharmacy adjacent to a small or medium-sized hospital (less than 400 beds) | 118 |  | 51 |  | 29 |  | 18 |  | 18 |  | 2 |
| Pharmacy located next to a clinic or medical office | 361 |  | 168 |  | 90 |  | 68 |  | 28 |  | 7 |
| Pharmacy located near (but not adjacent to) a clinic | 1 |  | 0 |  | 0 |  | 1 |  | 0 |  | 0 |
| Community-based pharmacy serving a wide range of prescriptions (not adjacent to a specific medical institution) | 78 |  | 34 |  | 22 |  | 12 |  | 9 |  | 1 |
| Pharmacy serving multiple medical institutions within a medical mall or similar complex | 89 |  | 39 |  | 15 |  | 16 |  | 15 |  | 4 |
| Pharmacy attached to a drugstore | 355 |  | 175 |  | 82 |  | 68 |  | 26 |  | 4 |
| No answer | 1 |  | 0 |  | 0 |  | 0 |  | 0 |  | 1 |
| Number of pharmacists working per day (full-time equivalent) | | | | | | | | | | | |
| 1 | 172 |  | 78 |  | 44 |  | 32 |  | 15 |  | 3 |
| 1.1-2 | 219 |  | 98 |  | 57 |  | 41 |  | 19 |  | 4 |
| 2.1-3 | 223 |  | 109 |  | 53 |  | 35 |  | 20 |  | 6 |
| 3.1-4 | 217 |  | 97 |  | 57 |  | 47 |  | 16 |  | 0 |
| 4.1-5 | 107 |  | 53 |  | 18 |  | 22 |  | 13 |  | 1 |
| >5.1 | 112 |  | 54 |  | 20 |  | 17 |  | 16 |  | 5 |
| Unknown | 4 |  | 4 |  | 0 |  | 0 |  | 0 |  | 0 |
| Gender | | | | | | | | | | | |
| Female | 613 |  | 295 |  | 134 |  | 110 |  | 61 |  | 13 |
| Male | 441 |  | 198 |  | 115 |  | 84 |  | 38 |  | 6 |
| Age group, years | | | | | | | | | | | |
| <30 | 197 |  | 100 |  | 52 |  | 31 |  | 14 |  | 0 |
| 30–39 | 456 |  | 232 |  | 102 |  | 81 |  | 34 |  | 7 |
| 40–49 | 228 |  | 97 |  | 57 |  | 52 |  | 20 |  | 2 |
| 50–59 | 126 |  | 47 |  | 27 |  | 21 |  | 24 |  | 7 |
| >60 | 47 |  | 17 |  | 11 |  | 9 |  | 7 |  | 3 |
| Continuous years in pharmacy practice | | | | | | | | | | | |
| <5 | 290 |  | 155 |  | 69 |  | 46 |  | 20 |  | 0 |
| 5–9 | 254 |  | 123 |  | 64 |  | 44 |  | 17 |  | 6 |
| 10–19 | 307 |  | 143 |  | 64 |  | 65 |  | 30 |  | 5 |
| >20 | 203 |  | 72 |  | 52 |  | 39 |  | 32 |  | 8 |
| Experience in nutritional counseling | | | | | | | | | | | |
| Never | 151 |  | 110 |  | 22 |  | 15 |  | 4 |  | 0 |
| Rarely | 428 |  | 231 |  | 115 |  | 53 |  | 26 |  | 3 |
| Occasionally | 310 |  | 104 |  | 78 |  | 80 |  | 40 |  | 8 |
| Sometimes | 151 |  | 47 |  | 32 |  | 45 |  | 23 |  | 4 |
| Frequently | 14 |  | 1 |  | 2 |  | 1 |  | 6 |  | 4 |
| Experience in responding to consultations regarding physical activity and exercise | | | | | | | | | | | |
| Never | 485 |  | 302 |  | 99 |  | 64 |  | 16 |  | 4 |
| Rarely | 297 |  | 125 |  | 89 |  | 44 |  | 36 |  | 3 |
| Occasionally | 193 |  | 52 |  | 46 |  | 65 |  | 25 |  | 5 |
| Sometimes | 74 |  | 13 |  | 15 |  | 20 |  | 20 |  | 6 |
| Frequently | 5 |  | 1 |  | 0 |  | 1 |  | 2 |  | 1 |
| Resistance to responding to consultations regarding physical activity and exercise | | | | | | | | | | | |
| Not at all reluctant | 150 |  | 62 |  | 37 |  | 22 |  | 20 |  | 9 |
| Not very reluctant | 405 |  | 160 |  | 108 |  | 78 |  | 51 |  | 8 |
| Neutral | 312 |  | 159 |  | 70 |  | 66 |  | 16 |  | 1 |
| Somewhat reluctant | 162 |  | 91 |  | 31 |  | 28 |  | 12 |  | 0 |
| Strongly reluctant | 25 |  | 21 |  | 3 |  | 0 |  | 0 |  | 1 |
| Experience in pharmacist-initiated support for physical activity and exercise | | | | | | | | | | | |
| Never | 268 |  | 201 |  | 41 |  | 20 |  | 5 |  | 1 |
| Rarely | 348 |  | 174 |  | 98 |  | 56 |  | 18 |  | 2 |
| Occasionally | 273 |  | 84 |  | 74 |  | 82 |  | 30 |  | 3 |
| Sometimes | 137 |  | 28 |  | 31 |  | 31 |  | 36 |  | 11 |
| Always | 28 |  | 6 |  | 5 |  | 5 |  | 10 |  | 2 |
| Intention to engage in pharmacist-initiated support for physical activity and exercise | | | | | | | | | | | |
| No | 108 |  | 79 |  | 16 |  | 10 |  | 3 |  | 0 |
| Yes | 946 |  | 414 |  | 233 |  | 184 |  | 96 |  | 19 |
| Experience in interprofessional collaboration in supporting physical activity and exercise | | | | | | | | | | | |
| Never | 835 |  | 431 |  | 188 |  | 136 |  | 67 |  | 13 |
| Rarely | 117 |  | 41 |  | 37 |  | 25 |  | 11 |  | 3 |
| Occasionally | 55 |  | 11 |  | 11 |  | 23 |  | 9 |  | 1 |
| Sometimes | 39 |  | 7 |  | 11 |  | 10 |  | 9 |  | 2 |
| Frequently | 8 |  | 3 |  | 2 |  | 0 |  | 3 |  | 0 |
| Belief: important for pharmacists to know patient’s frailty status | | | | | | | | | | | |
| Disagree | 4 |  | 3 |  | 0 |  | 1 |  | 0 |  | 0 |
| Somewhat agree | 17 |  | 11 |  | 6 |  | 0 |  | 0 |  | 0 |
| Neither agree or disagree | 102 |  | 59 |  | 20 |  | 21 |  | 2 |  | 0 |
| Agree | 574 |  | 304 |  | 142 |  | 95 |  | 32 |  | 1 |
| Strong agree | 357 |  | 116 |  | 81 |  | 77 |  | 65 |  | 18 |
| Belief: important for pharmacists to assess patient’s frailty status | | | | | | | | | | | |
| Disagree | 14 |  | 12 |  | 1 |  | 1 |  | 0 |  | 0 |
| Somewhat agree | 74 |  | 53 |  | 17 |  | 3 |  | 1 |  | 0 |
| Neither agree or disagree | 333 |  | 208 |  | 61 |  | 52 |  | 10 |  | 2 |
| Agree | 494 |  | 183 |  | 143 |  | 107 |  | 57 |  | 4 |
| Strong agree | 139 |  | 37 |  | 27 |  | 31 |  | 31 |  | 13 |
| Pharmacists support the physical activity and exercise of patients and pharmacy visitors | | | | | | | | | | | |
| Not necessary at all | 3 |  | 3 |  | 0 |  | 0 |  | 0 |  | 0 |
| Somewhat unnecessary | 37 |  | 18 |  | 13 |  | 4 |  | 2 |  | 0 |
| Neither necessary nor unnecessary | 211 |  | 122 |  | 43 |  | 36 |  | 10 |  | 0 |
| Somewhat necessary | 551 |  | 273 |  | 133 |  | 106 |  | 36 |  | 3 |
| Strongly necessary | 252 |  | 77 |  | 60 |  | 48 |  | 51 |  | 16 |
| Pharmacists provide patients and pharmacy visitors with support in nutritional guidance and oral care | | | | | | | | | | | |
| Not necessary at all | 5 |  | 3 |  | 2 |  | 0 |  | 0 |  | 0 |
| Somewhat unnecessary | 26 |  | 14 |  | 7 |  | 3 |  | 2 |  | 0 |
| Neither necessary nor unnecessary | 172 |  | 90 |  | 45 |  | 30 |  | 7 |  | 0 |
| Somewhat necessary | 559 |  | 277 |  | 137 |  | 99 |  | 43 |  | 3 |
| Strongly necessary | 292 |  | 109 |  | 58 |  | 62 |  | 47 |  | 16 |
| Pharmacists provide opportunities for local residents to engage in mutual interaction | | | | | | | | | | | |
| Not necessary at all | 16 |  | 6 |  | 7 |  | 2 |  | 0 |  | 1 |
| Somewhat unnecessary | 74 |  | 43 |  | 17 |  | 10 |  | 3 |  | 1 |
| Neither necessary nor unnecessary | 313 |  | 173 |  | 67 |  | 49 |  | 19 |  | 5 |
| Somewhat necessary | 452 |  | 207 |  | 113 |  | 85 |  | 42 |  | 5 |
| Strongly necessary | 199 |  | 64 |  | 45 |  | 48 |  | 35 |  | 7 |
| Recognition of the impact of medications on physical activity | | | | | | | | | | | |
| I don't know | 100 |  | 56 |  | 27 |  | 10 |  | 2 |  | 5 |
| No | 16 |  | 8 |  | 3 |  | 3 |  | 2 |  | 0 |
| Yes | 938 |  | 429 |  | 219 |  | 181 |  | 95 |  | 14 |
| Recognition of the concept of “rehabilitation pharmacotherapy” | | | | | | | | | | | |
| I haven't heard | 369 |  | 209 |  | 87 |  | 47 |  | 21 |  | 5 |
| I don't know | 541 |  | 247 |  | 136 |  | 102 |  | 45 |  | 11 |
| I know | 134 |  | 36 |  | 26 |  | 42 |  | 27 |  | 3 |
| I know well | 10 |  | 1 |  | 0 |  | 3 |  | 6 |  | 0 |
| Educational opportunities related to supporting physical activity and exercise | | | | | | | | | | | |
| Never | 832 |  | 436 |  | 190 |  | 135 |  | 59 |  | 12 |
| Completed a series | 105 |  | 30 |  | 30 |  | 31 |  | 10 |  | 4 |
| Several times in the past | 105 |  | 26 |  | 23 |  | 26 |  | 27 |  | 3 |
| Several times a year | 10 |  | 1 |  | 4 |  | 2 |  | 3 |  | 0 |
| No answer | 2 |  | 0 |  | 2 |  | 0 |  | 0 |  | 0 |
| Willingness to learn about supporting physical activity and exercise | | | | | | | | | | | |
| Not interested | 53 |  | 38 |  | 7 |  | 6 |  | 2 |  | 0 |
| If it became necessary | 318 |  | 189 |  | 71 |  | 40 |  | 17 |  | 1 |
| If the opportunity arises | 595 |  | 241 |  | 152 |  | 129 |  | 62 |  | 11 |
| Highly motivated | 88 |  | 25 |  | 19 |  | 19 |  | 18 |  | 7 |
| Willingness to learn about “rehabilitation pharmacotherapy” | | | | | | | | | | | |
| Not interested | 44 |  | 29 |  | 8 |  | 7 |  | 0 |  | 0 |
| If it became necessary | 282 |  | 169 |  | 56 |  | 40 |  | 16 |  | 1 |
| If the opportunity arises | 600 |  | 258 |  | 148 |  | 118 |  | 63 |  | 13 |
| Highly motivated | 128 |  | 37 |  | 37 |  | 29 |  | 20 |  | 5 |
| Values are presented as number unless otherwise indicated. | | | | | | | | |  |  |  |

**Table S3. Comparison of the characteristics of survey respondents with national statistics.**

|  | Overall | |  | National statistics | |
| --- | --- | --- | --- | --- | --- |
| Characteristics | N = 1,054 | 100.0% |  | N =197,437 | 100.0% |
| The seven regional divisions* | | |  |  |  |
| Hokkaido | 156 | 14.8% |  | 7,323 | 3.7% |
| Tohoku | 204 | 19.4% |  | 12,240 | 6.2% |
| Kanto | 378 | 35.9% |  | 74,494 | 37.7% |
| Chubu | 105 | 10.0% |  | 32,172 | 16.3% |
| Kinki | 169 | 16.0% |  | 34,229 | 17.3% |
| Chugoku-Shikoku | 21 | 2.0% |  | 16,499 | 8.4% |
| Kyusyu | 11 | 1.0% |  | 20,480 | 10.4% |
| Gender* | | |  |  |  |
| Female | 613 | 58.2% |  | 127,929 | 64.8% |
| Male | 441 | 41.8% |  | 69,508 | 35.2% |
| Age group, years* | | |  |  |  |
| <40 (<30 / 30–39) | 653 | 62.0% |  | 73,775 | 37.4% |
| >40 (40–49 / 50–59 / >60) | 401 | 38.0% |  | 123,662 | 62.6% |
| Values are presented as number (%) unless otherwise indicated. National statistics were calculated based on the reports [22] from the Ministry of Health, Labour and Welfare. Asterisk (*) indicates a statistically significant difference between this study data and national statistics (p < 0.05, chi-square test or Fisher’s exact test). | | | | | |

**Table S4. Sensitivity analysis of the associations between respondent characteristics and frailty assessment in pharmacy practice.**

| Characteristics | Crude odds ratio (95% CI) | Adjusted odds ratio (95% CI) |
| --- | --- | --- |
| Age group, years (ref = <40) | | |
| >40 | 2.02 (1.37–2.97) | 2.89 (1.86–4.49)* |
| Experience in nutritional counseling (ref = rarely or never) | | |
| Frequently/sometimes/occasionally | 3.60 (2.36–5.50) | 1.57 (0.95–2.57) |
| Experience in responding to consultations regarding physical activity and exercise (ref = rarely or never) | | |
| Frequently/sometimes/occasionally | 3.39 (2.29–5.02) | 1.19 (0.73–1.95) |
| Experience in pharmacist-initiated support for physical activity and exercise (ref = rarely or never) | | |
| Always/sometimes/occasionally | 6.03 (3.82–9.51) | 3.56 (2.06–6.14)* |
| Experience in interprofessional collaboration in supporting physical activity and exercise (ref = rarely or never) | | |
| Frequently/sometimes/occasionally | 2.80 (1.69–4.65) | 1.15 (0.62–2.12) |
| Belief: important for pharmacist to *assess* patient’s frailty status | | |
| Disagree/neither agree or disagree/somewhat agree/don’t know | 1 | 1 |
| Strong agree/agree | 6.24 (3.45–11.26) | 4.82 (2.52–9.20)* |
| Pharmacists support the physical activity and exercise of patients and pharmacy visitors | | |
| Not necessary at all/somewhat unnecessary/neither necessary nor unnecessary | 1 | 1 |
| Strongly necessary/somewhat necessary | 3.02 (1.63–5.60) | 1.19 (0.55–2.58) |
| Pharmacists provide patients and pharmacy visitors with support in nutritional guidance and oral care | | |
| Not necessary at all/somewhat unnecessary/neither necessary nor unnecessary | 1 | 1 |
| Strongly necessary/somewhat necessary | 3.16 (1.57–6.36) | 1.51 (0.64–3.55) |
| Pharmacists provide opportunities for local residents to engage in mutual interactions | | |
| Disagree/neither agree or disagree/somewhat agree/don’t know | 1 | 1 |
| Strongly agree/agree | 2.04 (1.31–3.16) | 1.01 (0.59–1.71) |
| Recognition of the concept of “rehabilitation pharmacotherapy” |  |  |
| I don’t know/I haven’t heard | 1 | 1 |
| I know well/I know | 3.36 (2.16–5.22) | 2.39 (1.43–4.00)* |
| Educational opportunities related to supporting physical activity and exercise (ref = never) | | |
| Several times a year/several times in the past/completed a series | 2.91 (1.94–4.36) | 1.64 (1.02–2.64)* |
| Willingness to learn about supporting physical activity and exercise (ref = not interested) | | |
| Highly motivated/if the opportunity arises/if it became necessary | 3.34 (0.80–13.91) | 0.56 (0.12–2.60) |
| Crude odds ratios and adjusted odds ratios (AORs) with 95% confidence intervals are presented. An odds ratio greater than 1 indicates a higher likelihood of conducting frailty assessment (Yes: “always,” “sometimes”; No: “occasionally,” “rarely,” or “never”) associated with the corresponding factor. AORs were estimated using multivariate logistic regression that included only variables found to be statistically significant (p < 0.05) in univariate analysis. Statistically significant AORs (p < 0.05) are marked with an asterisk (*). Reference categories are indicated in parentheses. | | |

**Supplemental Figure.**

**Figure S1. Co-occurrence networks of free-text responses to each question.**
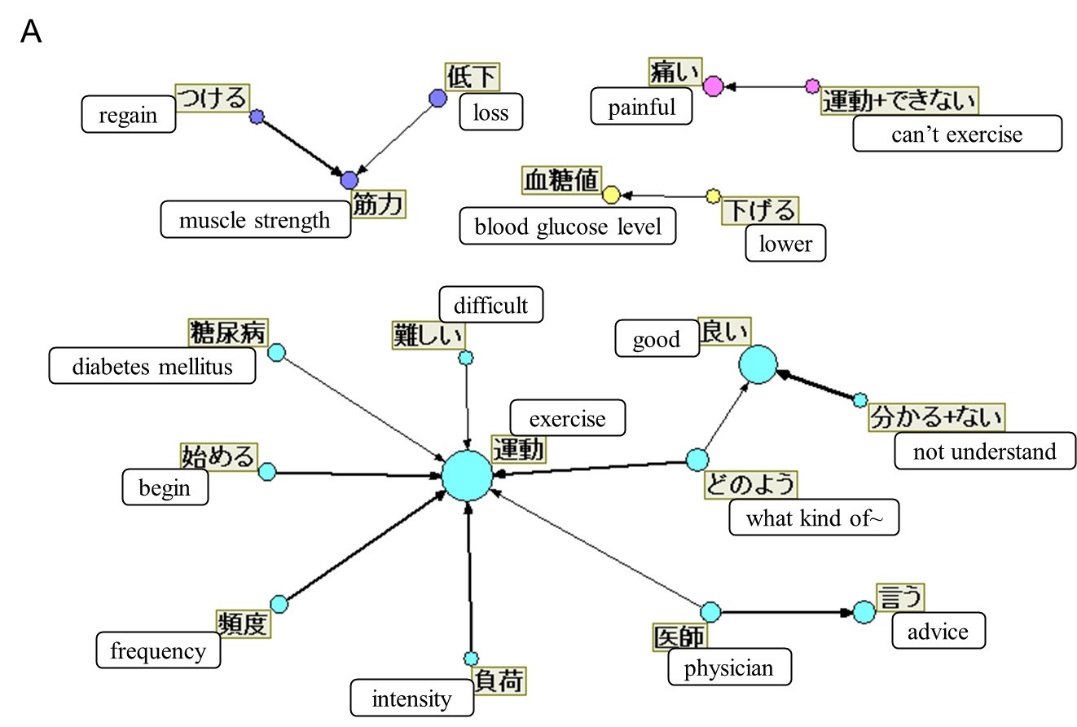


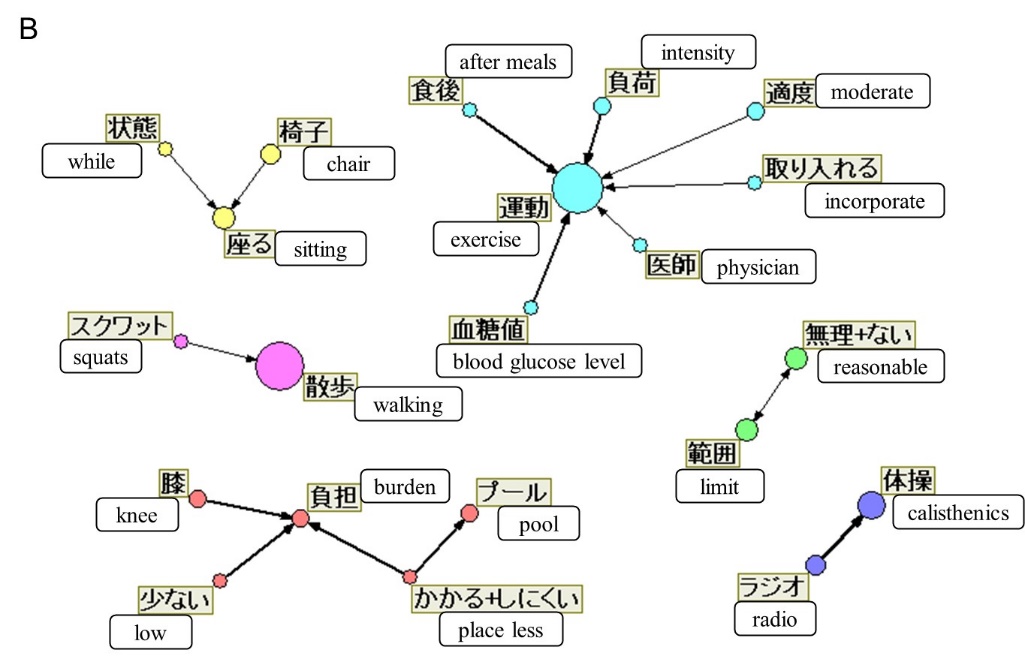


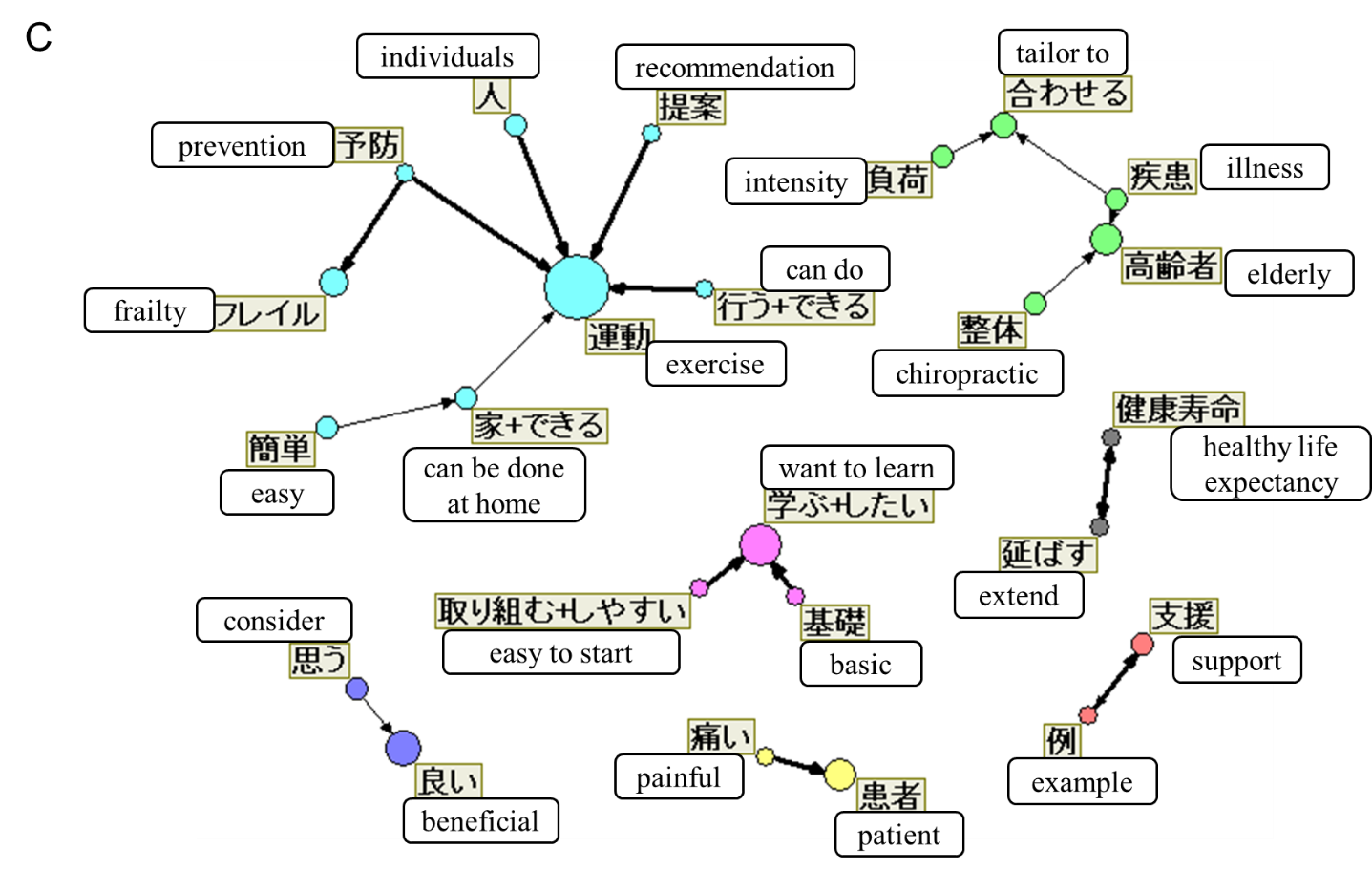


**Supplemental Figure legends**

**Figure S1. Co-occurrence networks of free-text responses to each question.**

The data shows the co-occurrence network of the descriptive responses to each question. (A) From the co-occurrence network related to consultation content, clusters of expressions such as “*I can't exercise because it is painful*,” “*What should I do to regain lost muscle strength?*” and “*I don't know what kind of exercise is good*” were extracted. (B) In pharmacist-initiated support activities, specific types of exercises such as “*radio calisthenics*,” “*squats and walking*,” and “*exercises while sitting in a chair*” were frequently mentioned, along with expressions showing consideration for patients or pharmacy visitors, such as “*exercises that do not burden the knees*” and “*exercises within reasonable limits*.” (C) Concerning topics they wish to learn, expressions such as “*appropriate intensity tailored to the elderly’s illnesses*,” “*frailty-preventive exercises that can be performed at home,*” and “*easy-to-start exercises*” were extracted, reflecting pharmacists’ interest in specific examples and approaches, as well as considerations for older adults and patients with chronic conditions.
